# CXCL3 contributes to CD133^+^ CSCs maintenance and forms a positive feedback regulation loop with CD133 in HCC via Erk1/2 phosphorylation

**DOI:** 10.1038/srep27426

**Published:** 2016-06-03

**Authors:** Lin Zhang, Lixing Zhang, Hong Li, Chao Ge, Fangyu Zhao, Hua Tian, Taoyang Chen, Guoping Jiang, Haiyang Xie, Ying Cui, Ming Yao, Jinjun Li

**Affiliations:** 1 State Key Laboratory of Oncogenes and Related Genes, Shanghai Cancer Institute, Renji Hospital, Shanghai Jiaotong University School of Medicine, Shanghai, China; 2 Cancer Research Institute, Fudan University Shanghai Cancer Center, Department of Oncology, Shanghai Medical College, Fudan University, Shanghai, China; 3 Qi Dong Liver Cancer Institute, Qi Dong, China; 4 Department of General Surgery, the First Affiliated Hospital, School of Medicine, Zhejiang University, Hangzhou, China; 5 Cancer Institute of Guangxi, Nanning, China

## Abstract

Although the chemotactic cytokine CXCL3 is thought to play an important role in tumor initiation and invasion, little is known about its function in hepatocellular carcinoma (HCC). In our previous study, we found that Ikaros inhibited CD133 expression via the MAPK pathway in HCC. Here, we showed that Ikaros may indirectly down-regulate CXCL3 expression in HCC cells, which leads to better outcomes in patients with CD133^+^ cancer stem cell (CSC) populations. CD133 overexpression induced CXCL3 expression, and silencing of CD133 down-regulated CXCL3 in HCC cells. Knockdown of CXCL3 inhibited CD133^+^ HCC CSCs’ self-renewal and tumorigenesis. The serum CXCL3 level was higher in HCC patients’ samples than that in healthy individual. HCC patients with higher CXCL3 expression displayed a poor prognosis, and a high level of CXCL3 was significantly associated with vascular invasion and tumor capsule formation. Exogenous CXCL3 induced Erk1/2 and ETS1 phosphorylation and promoted CD133 expression, indicating a positive feedback loop between CXCL3 and CD133 gene expression in HCC cells via Erk1/2 activation. Together, our findings indicated that CXCL3 might be a potent therapeutic target for HCC.

Hepatocellular carcinoma (HCC) is the sixth most common type of cancer and the third leading cause of cancer-related death worldwide^1^. Patients with early HCC achieve 5-year survival rates of approximately 70% with resection and liver transplantation, whereas patients with advanced HCC have a median survival of less than 1 year^2^. In recent decades, solid tumors have been found to be composed of a heterogeneous population of neoplastic cells; a small subset of cancer cells termed cancer stem cells (CSCs) may play a key role in tumor growth and recurrence^3^. Several cell surface proteins serve as CSC markers in HCC, including EpCAM, CD24, CD44, CD90 and OV6^4^,^5^. Our previous studies have shown that CD133^+^ HCC cells are of high tumorigenicity and chemotherapy resistance, with high expression of a number of stemness genes, and these cells could be induced to differentiate by exogenous BMP4 treatment, demonstrating that CD133 is also a CSC marker in HCC^6^,^7^,^8^.

Chemokines and their G-protein-coupled receptors were originally reported to mediate different pro- and anti-inflammatory responses^9^. Chemokines are subdivided into four families based on the position of the cysteine residues within the N-terminal region (CXC, CC, C and CX3C), and they exert their function by binding to their G-protein-coupled receptors, defined as, respectively, CXCR, CCR, CR or CX3CR. Chemokines play an essential role in tumor progression and act on endothelial, epithelial and tumor cells^10^. Shrivastava *et al.* found that CXCL1 and CXCL3 are significantly over-expressed during esophageal carcinogenesis^11^. Ding *et al.* reported that high CCL20 expression is associated with poor recurrence-free survival and overall survival, and CCL20 expression is an independent predictor of tumor recurrence^12^. Sutton *et al.* reported that CCL5 promotes metastasis and invasion of the HCC cell line Huh7 *via* the activation of FAK and MMP9^13^. Although many chemokines promote malignancy, CX3CL1 is believed to inhibit HCC tumor growth and recurrence^14^,^15^, suggesting that different chemokines may exert distinct functions in the same cancer.

CXCL3 is a member of the CXC chemokine family and is subclassified as a Glu-Leu-Arg (ELR^+^) CXC chemokine^16^. CXCL3 is over-expressed in most cases of aggressive prostate and breast tumors^17^,^18^. Luan *et al.* showed that CXCL3 is also an important mediator of tumor initiation in human melanoma^19^. In the liver, Simpson *et al.* reported that CXCL3 is widely expressed and is involved in liver injury and the inflammatory response^20^. Han *et al.* showed that CXCL3 was up-regulated in tumor tissue compared with its para-tumor tissue in a HCC xenograft model^21^. Our research showed that CXCL3 was significantly overexpressed in the CD133^+^ CSC population compared with its corresponding CD133^−^ non-CSC population, and CXCL3 expression was positively correlated with CD133 expression in HCC. The shRNA-mediated stable knockdown of CXCL3 inhibited CD133^+^ CSC proliferation and self-renewal *in vitro* and suppressed CD133^+^ HCC cell tumorigenesis *in vivo*, indicating that CXCL3 regulates the maintenance of stemness in CD133^+^ CSCs. Furthermore, we found that CXCL3 may be involved in a feedback loop regulating CD133 expression *via* the MAPK/ETS1 pathway in HCC and that HCC patients with higher CXCL3 expression levels displayed a poor prognosis.

## Results

### CXCL3 expression up-regulated in HCC cells with CD133 high-expression

In our previous study, we reported that Ikaros inhibited the expression of CD133 via direct binding to the CD133 P1 promoter and repressed the tumorigenic and self-renewal capacity of CD133^+^ CSCs. Decreased expression of Ikaros was significantly associated with poor survival in HCC patients^22^. Here, cDNA microarray analyses were performed, and we found that CXCL3 was down-regulated after Ikaros over-expression in HCC cells (Supplementary Table S1). Western blot results showed that Ikaros overexpression inhibited CXCL3 protein expression, and Ikaros knockdown induced CXCL3 expression upregulated in HCC cells (Supplementary Fig. S1A). However, although bioinformatics analysis of the CXCL3 promoter region showed one Ikaros binding site, ChIP assay demonstrated that Ikaros did not directly bind to this predicted site (Supplementary Fig. S1B). We then analyzed CXCL3 mRNA expression in HCC samples in the TCGA cohort. Kaplan-Meier survival analysis showed that patients with a high CXCL3 mRNA expression level had a poorer outcome. Investigation of the correlation between CXCL3 and clinicopathological features showed that a high level of CXCL3 was significantly associated with vascular invasion and TNM stage, and was negatively correlated with Hepatitis B virus infection (Supplementary Fig. S2 and Supplementary Table S2). Additional, Pearson correlation analysis result displayed that CXCL3 positively correlated with CD133 expression in the HCC tissues at the mRNA level (Supplementary Table S3). These results indicate that CXCL3 expression may be negatively correlated with the prognosis of HCC patients.

**Figure 1:**
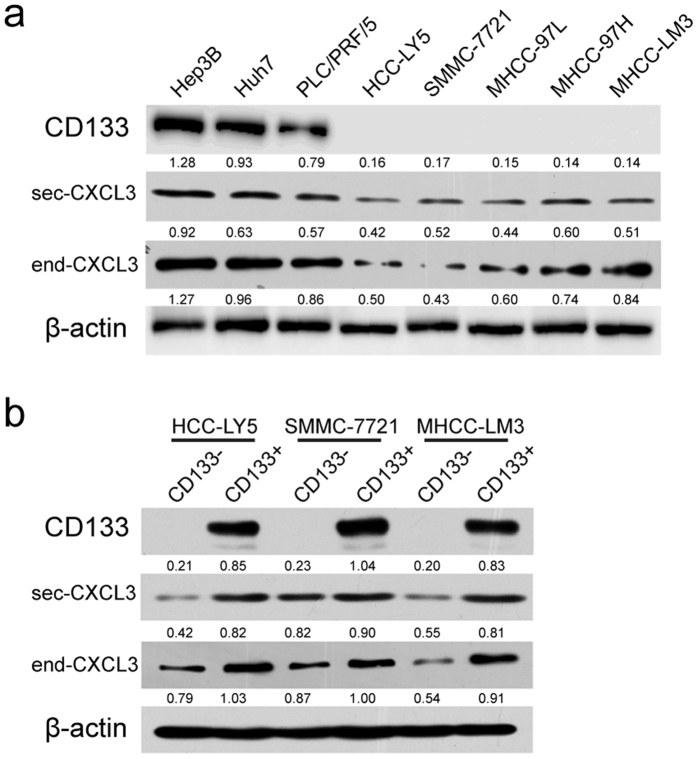
CXCL3 was differentially expressed in CD133^+^/CD133^−^ cell population of HCC cell lines. **(a**) Western blotting analysis of CD133, secreted and endogenous CXCL3 proteins expression in HCC cell lines. The gels in the same panel mentioned in this article had been run under the same experimental conditions. Uncropped full-length blots were showed in the Supplementary Fig. S4A. (**b**) CD133^+^ HCC cells expressed higher level of secreted and endogenous CXCL3 proteins compared with the corresponding CD133^−^ cells. Uncropped full-length blots were showed in the Supplementary Fig. S4B.

Western blotting assays showed that CXCL3 was differentially expressed in the 8 analyzed HCC cell lines, and a relatively high expression level of CXCL3 was observed in Hep3B, Huh7 and PLC/PRF/5 cells, which had high expression levels of CD133 (Fig. 1a)^7^,^8^. Then, CD133^+^ and CD133^−^ cells were sorted via MACS in three HCC cell lines, including HCC-LY5, SMMC-7721 and MHCC-LM3, which had low expression of CD133 (Supplementary Fig. S3A)^6^. In the CD133^+^ CSC population, CXCL3 expression was up-regulated compared with its corresponding CD133^−^ population (Fig. 1b). Because CXCL3 is a secretory protein, cell culture medium was collected, and secreted CXCL3 protein was analyzed. A similar increasing expression trend of secretory CXCL3 protein in the culture medium was confirmed by western blot (Fig. 1a,b), indicating that CXCL3 might regulate CSC CD133 expression.

### CXCL3 promotes the cell growth of CD133^+^ HCC CSCs

**Figure 2:**
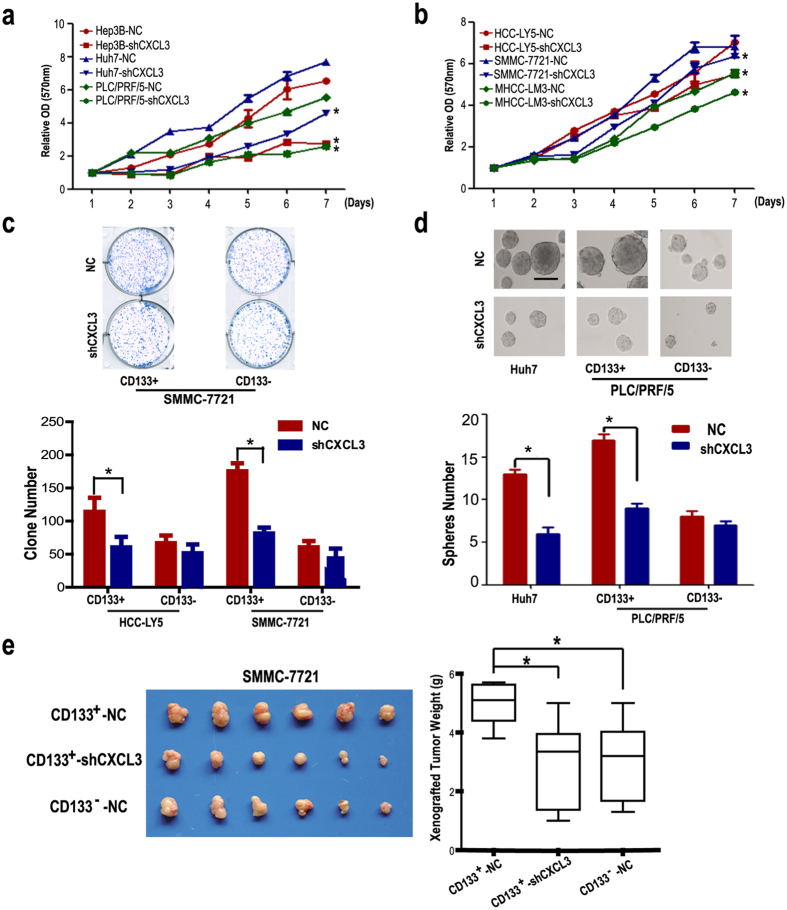
Down-regulation of CXCL3 expression inhibited HCC cells growth. (**a**) MTT assays showed that down-regulation of CXCL3 expression by shRNA inhibited cell growth in Hep3B, Huh7 and PLC/PRF/5 cells after day 4 (values were represented as the mean ± SD; *p < 0.05 *vs* shNC control, the Bonferroni method was used for the multiple comparison). (**b**) Down-regulation of CXCL3 inhibited cell growth in HCC-LY5, SMMC-7721 and MHCC-LM3 cells after day 4 (values were represented as the mean ± SD; *p < 0.05 *vs* shNC control, the Bonferroni method was used for the multiple comparison). (**c**) Results of the clone-formation assay in CXCL3 knockdown CD133^+^/CD133^−^ HCC cells sorted from HCC-LY5 (*p* = 0.0005) and SMMC-7721 (*p* = 0.028) (values were represented as the mean ± SD; *p < 0.05; *t*-test, *vs* cells treated with shNC). (**d**) Tumor-sphere formation analysis displayed that CXCL3-silence decreased the tumor-sphere number in Huh7 (*p* = 0.0325) and PLC/PRF/5 (*p* = 0.0491) CD133^+^ cells (values were represented as the mean ± SD; *p < 0.05; *t*-test, *vs* cells treated with shNC). (**e**) The weight of tumors from BALB/c (nu/nu) mice injected with CXCL3 knockdown or control SMMC-7721 cells are shown (CD133^+^-NC *vs* CD133^−^-NC, *p* = 0.045; CD133^+^-NC *vs* CD133^+^-shCXCL3, *p* = 0.037) (values were represented as the mean ± SD; *p < 0.05 *vs* cells treated with shNC, the Bonferroni method was used for the multiple comparison).

Our previous studies have shown that the CD133^+^ CSCs of HCC cell lines were distinctive for their high self-renewal and tumorigenesis capacities. Marotta *et al.* found that CXCL3 is required for growth in CD44^+^CD24^−^ human breast CSCs^23^. Four shRNA vectors were constructed, and the most efficient shRNA was used in the following assay (Supplementary Fig. S3B). The knockdown efficiency was confirmed by real-time RT-PCR (Supplementary Fig. S3C). MTT results showed that after CXCL3 stable knockdown, the cell growth was inhibited in HCC cell lines (either with CD133 high expression or with CD133 low expression) (Fig. 2a,b). The colony formation assay results demonstrated that CD133^+^ HCC cells possessed higher colony formation efficiency (CFE) than the corresponding CD133^−^ cells, and silencing of CXCL3 depressed the CFE of the CD133^+^ CSC population in HCC-LY5 and SMMC-7721 cells. However, the CFE of the CD133^−^ HCC cells was not significantly affected by the shCXCL3 treatment (Fig. 2c).

Self-renewal is an important feature of CSCs. Here, we used the tumor-sphere formation assay to analyze the self-renewal ability of HCC cells. Sorted CD133^+/−^ PLC/PRF/5 cells were cultured in suspension in CDM, and CXCL3 was then stably knocked down in both populations to observe their tumor-sphere formation ability. As shown in Fig. 2d, the tumor-spheres obtained from CD133^−^ cells were fewer in number and smaller than those formed from CD133^+^ cells. shCXCL3 treatment inhibited the tumor-sphere formation ability of CD133^+^ PLC/PRF/5 cells, and the tumor-sphere formation ability of CD133^−^ PLC/PRF/5 cells was also impaired after CXCL3 knockdown, though it was not so obvious compared with CD133^+^ cell group. Similar results were observed in non-sorted Huh7 cells. We then analyzed the stemness-related genes expression after CXCL3 knockdown. Realtime PCR results displayed that stemness-related genes including Oct4, EP300, Tert and β-catenin were downregulated in Huh7-shCXCL3 cells (Supplementary Fig. S3B). Exogenous CXCL3 treatment induced stemness-related genes including Oct4, EP300, Tert and β-catenin expression in SMMC-7721 cells, indicating that CXCL3 may contribute to stemness-related genes expression in HCC cells (Supplementary Fig. S3D).

The tumorigenicity of CD133^+/−^ cells with CXCL3 stable knockdown was analyzed in an immunodeficient mouse xenograft model. To ensure that xenograft tumors formed, a large cell number was used. As we reported previously, CD133^+^ PLC/PRF/5 cells developed larger tumors compared with the corresponding CD133^−^ cells. CXCL3 silencing inhibited tumor growth in the CD133^+^ population, although there was no significant difference in the tumor mass between CD133^+^-shCXCL3 and CD133^−^ cells groups (Fig. 2e), suggesting that CXCL3 promoted CD133^+^ HCC CSC maintenance *in vivo*.

### Exogenesis CXCL3 induces CD133 upregulation via Erk1/2 phosphorylation

**Figure 3:**
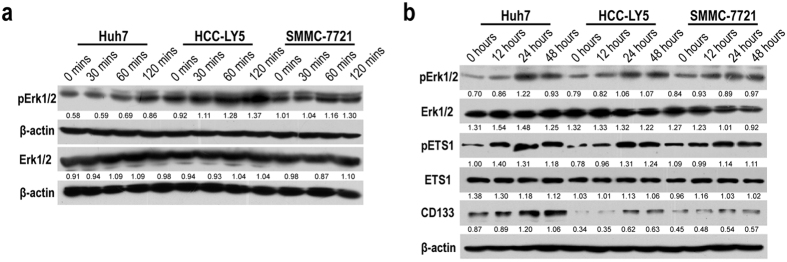
Exogenous CXCL3 treatment induced Erk/MAPK pathway activation. (**a**) 100 ng/ml exogenous CXCL3 treatment induced Erk1/2 phosphorylation in a time dependent manner in Huh7, HCC-LY5 and SMMC-7721 cells. Uncropped full-length blots were showed in the Supplementary Fig. S5A. (**b**) Long time treatment of CXCL3 (100 ng/ml) induced Erk1/2 and EST1 phosphorylation, and promoted CD133 expression in HCC cells. Uncropped full-length blots were showed in the Supplementary Fig. S5B.

Recent report showed that the airway smooth muscle cell migration induced by CXCL3 depends on the Erk1/2 MAPK pathway via CXCR2^24^. And Erk1/2 phosphorylation inhibitor U0126 and PD98059 treatment depressed CD133 expression in HCC cells^22^. Considering that CXCL3 may exert different effect on MAPK activation in HCC cells, we incubated HCC cells with 100 ng/ml CXCL3 for different periods of time. Erk1/2 phosphorylation was gradually induced in a time-dependent way (Fig. 3a). Long-term exposure to CXCL3 increased phosphorylation of both Erk1/2 and ETS1 in Huh7, HCC-LY5 and SMMC-7721 cells. Western blotting analysis also showed that CD133 protein expression was induced in these CXCL3-treated HCC cells (Fig. 3b).

### CXCL3 expression positively correlates with CD133 expression

**Figure 4:**
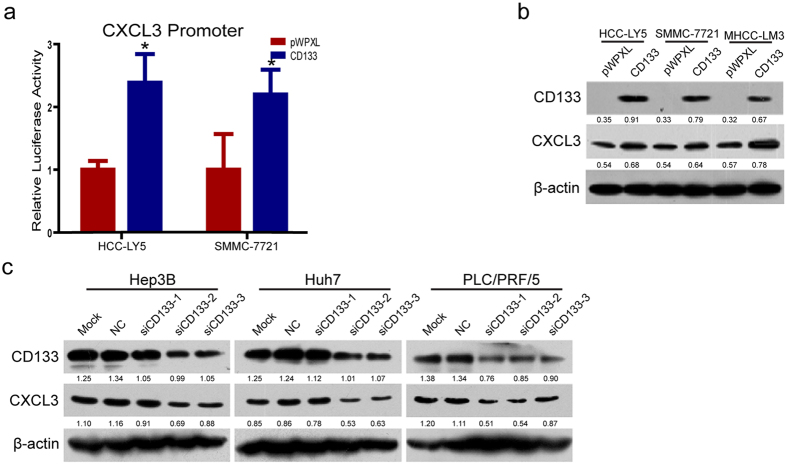
CXCL3 expression positively correlated with CD133 expression in HCC cells. (**a**) CD133 was overexpressed in HCC-LY5 and SMMC-7721 cells and the luciferase activities associated with CXCL3 promoter are shown. Reporter gene activities are expressed as fold changes relative to the control (*p* = 0.002 in HCC-LY5; *p* = 0.003 in SMMC-7721) (values were represented as the mean ± SD; *p < 0.05; *t*-test, *vs* vector control). (**b**) CD133 overexpression up-regulated CXCL3 protein expression in HCC cell lines. Uncropped full-length blots were showed in the Supplementary Fig. S6A. (**c**) CD133 knockdown down-regulated CXCL3 expression in HCC cells. Uncropped full-length blots were showed in the Supplementary Fig. S6B–D.

To explore whether CD133 could up-regulate CXCL3 expression, CXCL3 expression was detected after CD133 ectopic over-expression or silencing in HCC cells. As shown in Fig. 4a, the luciferase reporter assay demonstrated that the CXCL3 promoter activity was increased in HCC-LY5 and SMMC-7721 cells that overexpressed CD133. Western blot results showed that CD133 over-expression promoted endogenous CXCL3 expression in HCC-LY5, SMMC-7721 and MHCC-LM3 cells (Fig. 4b). siRNA oligonucleotides specifically targeting CD133 were synthesized, and the knockdown efficiency was reported previously^22^. As shown in Fig. 4c, knock-down of CD133 in Hep3B cells leaded to CXCL3 expression down-regulation. Similarly results could be obtained in Huh7 and PLC/PRF/5 cells (Fig. 4c). These results indicate that there might be a positive feedback regulation loop between CD133 and CXCL3 expression in HCC cells.

### High CXCL3 expression level indicates poor survival in HCC patients

**Figure 5:**
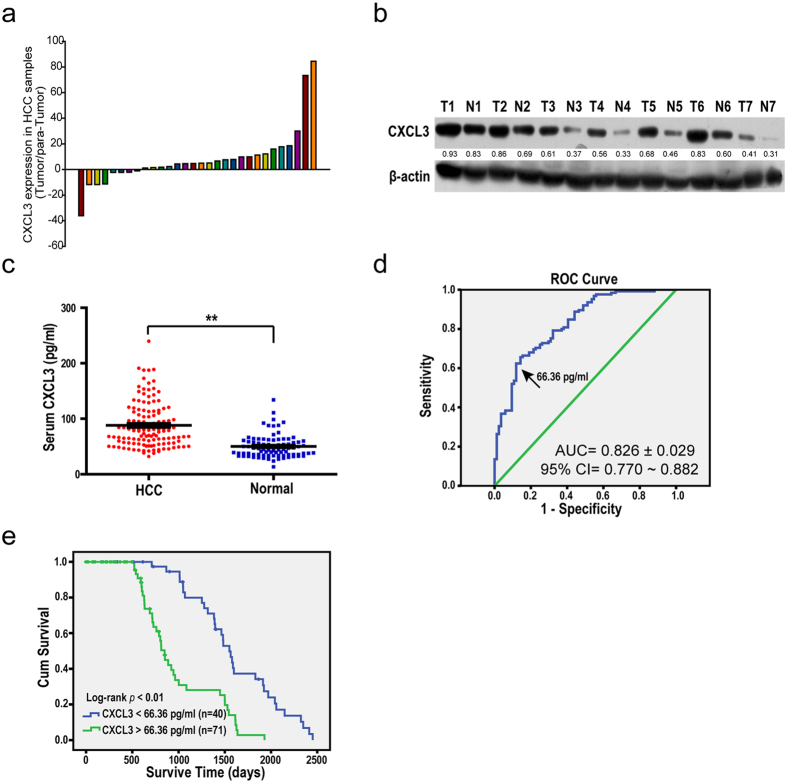
HCC patients with high CXCL3 expression level associated with unfavorable prognosis. (**a**) Realtime PCR analysis demonstrated that HCC tumor tissue samples expressed higher level of CXCL3 mRNA (22/30) compared with the para-tumor tissue samples. (**b**) Western blotting analysis of 7 pair tissue samples showed that CXCL3 HCC tumor tissue samples (T) expressed higher level of CXCL3 protein compared with the para-tumor tissue samples (N). Uncropped full-length blots were showed in the Supplementary Data. (**c**) ELISA results displayed that serum CXCL3 lever was higher in HCC patients (*p* = 0.0418)(values were represented as the mean ± SD; **p < 0.01; *t*-test, *vs* normal people control). (**d**) The cutoff value of 66.36 pg/ml for serum CXCL3 with a sensitivity of 62.4% and specificity of 88.1% using receiver operating characteristic analysis. (**e**) Results of overall survival analysis of serum CXCL3 in HCC patients are shown (Kaplan-Meier analysis).

We analyzed CXCL3 mRNA expression in 30 HCC patients’ tissue and para-tumor tissue samples. Real-time PCR results indicated that tumor tissue samples expressed a higher level of CXCL3 compared with the corresponding adjacent non-cancerous tissue samples (Fig. 5a). Western blot analysis indicated a similar expression pattern at the protein level (Fig. 5b).

Because CXCL3 is a secretory protein and plays an important role in HCC cells *in vitro*, we detected and compared serum CXCL3 protein levels in 125 HCC patients and 90 healthy individuals. The serum CXCL3 level was significantly higher in HCC patients’ samples than in healthy controls (Fig. 5c). A cutoff value of serum CXCL3 (66.36 pg/ml), which was of a sensitivity of 62.4% and a specificity of 88.1% (Fig. 5d, Area Under Curve is 0.826 ± 0.029, with the 95% CI of 0.770 to 0.882), was used to divide 125 HCC patients into two groups: 47 (37.6%) patients in the low CXCL3 expression group, and 78 (62.4%) patients in the high CXCL3 expression group. As shown in Fig. 5e, the overall survival analysis revealed that the high expression of CXCL3 in the serum was closely associated with poor outcomes in HCC patients, and patients with low CXCL3 expression levels were more likely to have a capsule around the tumor tissue and possess no vascular invasion (Table 1). These results suggest that CXCL3 is a candidate prognostic biomarker for HCC.

## Discussion

Persistent inflammation promotes and exacerbates malignancy. HCC is a clear example of an inflammation-related cancer, as more than 90% of HCCs arises in the context of hepatic inflammation, which involves the activation of a complex cytokine and chemokine network^25^. Chemokines play a role in many biological events, such as embryonic development, wound healing, angiogenesis, and most importantly, inflammatory diseases^10^. Some chemokines actively participate in the initiation, promotion and progression of tumors.

The CXC chemokines are heparin-binding proteins, with a heparin-binding domain in the C-terminus of the molecule, which displays disparate roles in the regulation of angiogenesis^26^. The N-terminus of several CXC chemokines contains three amino acid residues (ELR motif), and ELR^+^ members are angiogenic factors and potent neutrophil chemoattractants. In contrast, ELR^−^ CXC chemokines are potent inhibitors of angiogenesis and attract mononuclear leukocytes^27^,^28^. In the tumor context, the angiogenic activity could be considered as tumor-promoting, and several ELR^+^ CXC chemokines promote tumor growth^29^. The ELR^+^ chemokines CXCL1, CXCL2 and CXCL3 play roles in the growth of pancreatic cancer, melanoma, lung cancer and gastric cancer^30^,^31^,^32^,^33^. CXC chemokines are also involved in several types of CSC growth. CXCL12 (SDF1) is the best investigated chemokine among the CSC studies, and it promotes glioma stem cell proliferation and regulates the glioblastoma microenvironment^34^,^35^. The CXCL12-CXCR4 axis regulates the growth and invasion of ovarian CSCs^36^,^37^. Marotta *et al.* reported that CXCL3 is required for cell growth or proliferation in CD44^+^CD24^−^ human breast CSCs and induces STAT3 activation^23^.

In our present study, we found that the CXCL3 was overexpressed in the CD133^+^ HCC CSC population compared with its corresponding CD133^−^ cells. Knockdown of CXCL3 inhibited CD133^+^ HCC CSC self-renewal and tumorigenesis. Kogan-Sakin *et al.* reported that CXCL3 secretion from prostate stromal cells is induced by prostate epithelial cells, and this interaction contributes to the development of prostate cancer, revealing a role of secreted CXCL3 in tumorigenesis^38^. We analyzed the secreted CXCL3 in the culture medium and patients’ serum samples and found that it was also up-regulated in the CD133^+^ CSC group culture medium *in vitro*, demonstrating that CXCL3 may regulate CD133^+^ CSC stemness in an autocrine way. The ELISA assay showed that the serum CXCL3 levels were higher in HCC patients compared with healthy individuals, and HCC patients with higher CXCL3 expression displayed a poorer prognosis. A high CXCL3 expression level was positively correlated with vascular invasion and TNM stage, indicating that CXCL3 might be a potent therapeutic target for HCC. Although CXCL3 mRNA expression was negatively correlated with HBV infection in the TCGA cohort, the serum CXCL3 analysis showed that patients with low serum CXCL3 levels were more likely to have a capsule around the tumor, and CXCL3 expression was positively correlated with the percentage of stromal cells at the top of the HCC tumor (TCGA cohort, Supplementary Fig. S2B), suggesting a relationship between CXCL3 and inflammation phenomenon in HCC tissue. However, the underlying mechanism needs to be verified in our future investigations. Notably, the clinicopathologic CXCL3 expression levels were not exactly matched at the mRNA and protein levels, which may be due partly to the different genetic backgrounds of the HCC patients, and the serum CXCL3 level was more susceptible to the physical condition of the individual patient.

Ikaros is a member of the Kruppel-like family of zinc finger DNA-binding proteins. It functions mainly as a key regulator of early B cell development^39^. Loss of Ikaros function may be essential to the development of lymphoid leukemia^40^. In solid cancer, our previous results showed that decreased Ikaros expression was significantly associated with poor survival in HCC patients. Ikaros expression is up-regulated by ETS1, and its activity is regulated by the MAPKs pathway^22^. Here, we showed that Ikaros down-regulated CXCL3 expression in HCC cells, although Ikaros did not directly bind to its DNA sequence. Interestingly, we found that CD133 overexpression promoted CXCL3 expression in HCC cells, whereas CD133 knockdown inhibited CXCL3 expression, indicating that CXCL3 is also a downstream target of CD133. Although several regulation patterns of CD133 expression have been reported, the molecular function of CD133 remains unclear. Bourseau-Guilmain *et al.* reported that CD133 knockdown could enhance colon cancer cells transferring endocytosis^41^. Shimozato *et al.* reported that the phosphorylation status of the CD133 protein plays a role in colon cancer cell tumorigenesis. PTPRK can bind to CD133 protein, catalyze dephosphorylation of CD133 and abrogate CD133-mediated AKT phosphorylation^42^. These studies indicate that CD133 can regulate cell metabolism and be involved in cell signaling pathways through its intracellular domains. Although we observed that CD133 regulates CXCL3 expression in HCC cell lines, the regulatory mechanism of CXCL3 on CD133 expression in HCC still needs further investigation. However, We speculated that CD133 function as a membrane protein and might regulate down-stream signal pathway activity and relative transcription factors, and which affects the CXCL3 promoter activity and increases CXCL3 expression.

The CXCL3 receptor CXCR2 has been identified as a key mediator of neutrophil-associated inflammation in liver, and expression levels of CXCR2 are significantly increased in HCC^43^. CXCL3 activates CXCR2 through several signaling pathways, including MAPK and JAK2/STAT3^23^,^24^. Previous studies have found that MAPK signaling was activated in the CD133^+^ cancer cells in colon cancer and HCC^44^,^45^. Rohani *et al.* reported that PAR1 and PAR2 activated MAPK signaling and induced CXCL3 and CXCL5 expression in oral keratinocytes^46^. Bandow *et al.* reported that LPS could induce CXCL3 expression via Cot/Tpl2-ERK axis in macrophages^47^. We found that exogenous CXCL3 treatment induced Erk1/2 phosphorylation in HCC cells and promoted CD133 expression in a time-dependent manner. Erk1/2 activation induced ETS1 phosphorylation and inhibited the expression of its downstream gene, Ikaros, which contributed to CD133 up-regulation, indicating that a positive feedback loop between CD133 and CXCL3 expression regulation with MAPK signaling involved. The mechanism behind this phenomenon needs further investigation.

CXCL3 expression was up-regulated in the HCC tumor tissues, and HCC patients with higher CXCL3 expression displayed a poorer prognosis. CXCL3 plays a critical role in CD133^+^ CSC maintenance and might be a potent therapeutic target for HCC. CXCL3 expression is positively correlated with CD133 *in vitro* and *in vivo.* CXCL3 forms a positive feedback regulation loop with CD133 via MAPK signaling. However, the mechanism by which CD133 regulates CXCL3 expression needs further investigation.

## Materials and Methods

### Cell lines

PLC/PRF/5 and Hep3B were obtained from the American Type Culture Collection (Manassas, VA, USA). SMMC-7721 cells were obtained from the Cell Bank of the Institute of Biochemistry and Cell Biology, China Academy of Sciences (Shanghai, China). Huh7 cells were purchased from the Riken Cell Bank (Tsukuba, Japan). MHCC-LM3 cells were provided by the Liver Cancer Institute of Zhongshan Hospital, Fudan University (Shanghai, China). HCC-LY5 was established from primary HCC tissue in our lab. All cell lines were cultured in DMEM (Sigma-Aldrich, St. Louis, Missouri, USA) supplemented with 10% bovine serum (Hyclone, Logan, Utah, USA) and incubated in 5% CO_2_ at 37 °C. For the *in vitro* tumor-sphere formation assay with CSCs, single HCC cells were cultured suspension in Ultra low attachment multiwell plates (Costar, St. Louis, Missouri, USA) in the conditional medium (CDM). The CDM consisted of DMEDM/F12 supplemented with 0.5 × B27 supplement, 10 ng/ml basic fibroblast growth factor (bFGF) (Millipore, Billerica, Massachusetts, USA) and 10 ng/ml epithelial growth factor (EGF) (Millipore, USA).

### Chromatin immunoprecipitation (ChIP)

The cells were cross-linked with 10% paraformaldehyde at 37 °C for 10 minutes, and crosslinking was reversed with treatment with 1 M glycine for 5 min. After washing with 1× PBS buffer, the cells were harvested in T-PER Tissue Protein Extraction Reagent (Thermo Scientific, New York, USA) for 5 minutes on ice and centrifuged at 2,000 *g* for 5 minutes. The precipitants were suspended in nuclei lysis buffer, and the DNA was shredded into fragments of 1,000 base pairs by sonication. Antibodies against Ikaros (Santa Cruz Biotechnology, Dallas, Texas, USA) with protein G agarose beads (Sigma-Aldrich, USA) were added, and the samples were incubated overnight at 4 °C. After reversing the crosslinks, the DNA was isolated and used for polymerase chain reaction (PCR) analysis. The primers for qPCR are listed in Supplementary Tables S4 and S5.

### Western blotting

Western blotting was performed as described by the SuperSignal West Femto Maximum Sensitivity Substrate Kit (Pierce, New York, USA). Considering that the endogenous CXCL3 expression was relatively low in the HCC cell lines and patient tissues, the total protein samples loaded for Western Blot analysis was add to a certain amount in this study. The primary and HRP-conjugated secondary antibodies are listed in Supplementary Table S6.

### Plasmid constructs, lentivirus production, and cell transfection

Full-length human CD133 was subcloned into pWPXL (Addgene, Cambridge, Massachusetts, USA) plasmid as previously described^22^. A generic negative control (NC) sequence was synthesized by GenePharma (Shanghai, China). CXCL3 and NC shRNA were subcloned into pLVTHM (Addgene, USA) as described on the Addgene website. Virus packaging and cell transfection were performed as previously described^22^. Primers for cloning and the CXCL3 promoter sequence are provided in Supplementary Tables S7–S9.

### Plate colony formation assay

Two thousand cells were seeded into six-well culture plates. Cells were cultured under normal condition, and fixed 10~14 days later using 10% formaldehyde for 30 minutes at 37 °C. Cell colonies were stained with GIEMSA (Sigma-Aldrich, USA) for 30 minutes. After washing, the cell colonies were quantified.

### MTT assay

Three thousand cells were seeded into 96-well culture plates and incubated for the indicated time. Next, 100 μL MTT (5 mg/ml) was added to each well. Cells were incubated for 4 hours at 37 °C. The medium were carefully removed, and 100 μL DMSO was added. The OD value was recorded at an absorbance of 570 nm.

### Magnetic-activated cell sorting (MACS)

PLC/PRF/5, HCC-LY5, SMMC-7721, and MHCC-97L cells were magnetically isolated from CD133^+^ and CD133^−^ cells with the PE-conjugated anti-human CD133/1 antibody (AC133, Miltenyi Biotec, Bergisch Gladbach, Germany) using the Easystep PE Selection Kit (StemCell Technologies, British Columbia, Canada) according to the manufacturer’s instructions.

### Luciferase reporter assay

Cells were plated in 96-well culture plates for 24 hours and transfected with the appropriate constructs. Renilla and firefly luciferase activities were measured according to the manufacturer’s instructions (Promega, Madison, Wisconsin, USA).

### Bioinformatics analysis

A total of 355 liver cancer patients with at least a 5-year follow-up from The Cancer Genome Atlas (TCGA, https://tcga-data.nci.nih.gov/tcga/, updated at the end of December 31, 2014) database (TCGA cohort) were enrolled in this study for survival analyses and analyses of the relationship of CXCL3 mRNA expression with clinicopathological features.

Transcription factor binding sites in the CXCL3 promoter region were predicted using the TFSEARCH database (http://www.gene-regulation.com/pub/databases.html).

### Enzyme-linked immunosorbent Assay (ELISA)

ELISA was performed with the Human C-X-C motif chemokine 3 (CXCL3) ELISA Kit according to the manufacturer’s instructions (CUSABIO, Wuhan, Hubei, China). The serum samples were diluted 1:10 in Sample Diluent. In total, 125 HCC patients (125 cohort) and 90 healthy individuals with serum samples were tested.

For the use of clinical materials for research purposes, the procedure of human sample collection was approved by the Ethics Committee of Renji Hospital, Shanghai Jiao Tong University School of Medicine. All patients signed informed consent for the collection and use of their serum samples for this study. The experiments were performed in accordance with approved guidelines of Shanghai Jiao Tong University School of Medicine.

### Tumor xenograft models

Six- to eight-week-old male BALB/c (nu/nu) mice were randomly divided into groups and inoculated with suspended HCC cells (2 × 10^6^ cells/mouse). After observation for 4–6 weeks, the animals were sacrificed. Immediately after killing, xenograft tumors were weighed and fixed in neutral buffered formalin.

All animal experimental protocols were approved by the Institutional Animal Care and Use Committee of the Shanghai Jiao Tong University School of Medicine and the experiments were carried out in accordance with the approved guidelines. The animals were treated humanely according to the institutional animal care guidelines.

### Statistical analysis

Statistical analyses were performed using SPSS 13.0 software. The results are presented as the mean ± SD and compared using Student’s *t*-test. *p* < 0.05 was considered significant (*p < 0.05, **p < 0.01).

## Additional Information

**How to cite this article**: Zhang, L. *et al.* CXCL3 contributes to CD133^+^ CSCs maintenance and forms a positive feedback regulation loop with CD133 in HCC via Erk1/2 phosphorylation. *Sci. Rep.* **6**, 27426; doi: 10.1038/srep27426 (2016).

## Supplementary Material

Supplementary Data

## Figures and Tables

**Table 1:** Correlation between Clinicopathologic Features and Serum CXCL3 Protein Expression.

		CXCL3		*p* Value
		Low	High	
		Count	Count	
Gender	Male	35 (74.47%)	58 (74.36%)	0.989
	Female	12 (25.53%)	20 (25.64%)	
Age	<50	21 (44.68%)	36 (46.15%)	0.873
	>50	26 (55.32%)	42 (53.85%)	
AFP	<200 pg/ml	29 (65.91%)	50 (64.10%)	0.841
	>200 pg/ml	15 (34.09%)	28 (35.90%)	
Intrahepaticmetastasis	No	30 (63.83%)	55 (70.51%)	0.438
	Yes	17 (36.17%)	23 (29.49%)	
Vascular Invasion	No	40 (100%)	68 (90.67%)	0.046^a^*
	Yes	0 (0%)	7 (9.33%)	
Capsule	No	10 (21.28%)	36 (46.15%)	0.005**
	Yes	37 (78.72%)	42 (53.85%)	
Histology grade	I + II	21 (48.84%)	36 (47.39%)	0.878
	III + IV	22 (51.16%)	40 (52.61%)	
Tumor	<3cm	20 (47.62%)	40 (52.63%)	0.602
	>3cm	22 (52.38%)	36 (47.37%)	

^a^More than 20% of cells in this subtable have expected cell counts less than 5. Chi-square results may be invalid; *The Chi-square statistic is significant at the 0.05 level; **The Chi-square statistic is significant at the 0.01 level.
