# Supplementary material for: CXCL3 contributes to CD133+ CSCs maintenance and forms a positive feedback regulation loop with CD133 in HCC via Erk1/2 phosphorylation
Source: Sci Rep. 2016 Jun 3;6:27426. doi: 10.1038/srep27426 (PMC4891684; doi:10.1038/srep27426)
Supplement: Supplementary Data [file srep27426-s1.doc]

**CXCL3 contributes to CD133^+^ CSCs maintenance and forms a positive feedback regulation loop with CD133 in HCC cells via Erk1/2 phosphorylation**

Zhang Lin^1,2#^, Lixing Zhang^1#^, Hong Li^1^, Chao Ge^1^, Fangyu Zhao^1^, Hua Tian^1^, Taoyang Chen^3^, Guoping Jiang^4^, Haiyang Xie^4^, Ying Cui^5^, Ming Yao^1^, Jinjun Li^1*^

*^1^ State Key Laboratrory of Oncogenes and Related Genes, Shanghai Cancer Institute, Renji Hospital, Shanghai Jiaotong University School of Medicine, Shanghai, China*

*^2^ Cancer Research Institute, Fudan University Shanghai Cancer Center, Department of Oncology, Shanghai Medical College, Fudan University, Shanghai, China*

*^3^ Qi Dong Liver Cancer Institute, Qi Dong, China*

*^4^ Department of General Surgery, the First Affiliated Hospital, School of Medicine, Zhejiang University, Hangzhou, China*

*^5^ Cancer Institute of Guangxi, Nanning, China*

***^#^ These authors contributed equally to this work.***

**Supplementary Results**

**Supplementary Table S1**  List of differential expression genes in Huh7 HCC cells overexpressing Ikaros or pWPXL vector.

| Gene Symbol | Gene Tile | UniGene ID | Relative Fold Change |
| --- | --- | --- | --- |
| **Upregulated genes** | | | |
| IKZF1 | IKAROS family zinc finger 1 (Ikaros) | Hs.435949 | 365.085 |
| TNFSF10 | tumor necrosis factor (ligand) superfamily, member 10 | Hs.478275 | 21.1033 |
| IGFBP3 | insulin-like growth factor binding protein 3 | Hs.450230 | 13.6356 |
| TP53INP1 | tumor protein p53 inducible nuclear protein 1 | Hs.492261 | 10.8649 |
| GFI1 | growth factor independent 1 transcription repressor | Hs.73172 | 7.9077 |
| BMF | Bcl2 modifying factor | Hs.591104 | 7.7668 |
| ARPP-21 | cyclic AMP-regulated phosphoprotein, 21 kD | Hs.475902 | 5.1209 |
| DNMT3A | DNA (cytosine-5-)-methyltransferase 3 alpha | Hs.515840 | 4.2298 |
| PTEN | phosphatase and tensin homolog | Hs.500466 | 2.7457 |
| BCL2L11 | BCL2-like 11 (apoptosis facilitator) | Hs.469658 | 2.5003 |
| HDAC4 | histone deacetylase 4 | Hs.20516 | 2.0123 |
| **Downregulated genes** | | | |
| IL8 | interleukin 8 | Hs.624 | 0.0306 |
| TGFB1I1 | transforming growth factor beta 1 induced transcript 1 | Hs.513530 | 0.0324 |
| ABCC2 | ATP-binding cassette, sub-family C (CFTR/MRP), member 2 | Hs.368243 | 0.1099 |
| **CXCL3** | **chemokine (C-X-C motif) ligand 3** | **Hs.89690** | **0.186** |
| PROM1 | prominin 1 (CD133) | Hs.614734 | 0.1874 |
| FOSL2 | FOS-like antigen 2 | Hs.220971 | 0.2749 |
| ARHGAP18 | Rho GTPase activating protein18 | Hs.486458 | 0.3009 |
| CXCR7 | chemokine (C-X-C motif) receptor 7 | Hs.471751 | 0.3586 |
| MMP14 | matrix metallopeptidase 14 (membrane-inserted) | Hs.2399 | 0.3742 |
| JUN | jun oncogene | Hs.714791 | 0.3877 |
| NOTCH2 | Notch homolog 2 (Drosophila) | Hs.487360 | 0.4117 |
| GATA6 | GATA binding protein 6 | Hs.514746 | 0.4309 |
| EPCAM | epithelial cell adhesion molecule | Hs.542050 | 0.4498 |

**Supplementary Table S2.** Correlation between Clinicopathologic Features and CXCL3 mRNA Expression in TCGA Cohort

|  | CXCL3_IND | | |  |
| --- | --- | --- | --- | --- |
|  |  | Low | High |  |
|  |  | Count | Count | *p* Value |
| Gender | Male | 142 (69.27%) | 98 (65.33%) | 0.434 |
|  | Female | 63 (30.73%) | 52 (34.67%) |  |
|  |  |  |  |  |
| Hepatitis B | No | 137 (64.93%) | 126 (79.25%) | 0.003** |
|  | Yes | 74 (35.07%) | 33 (20.75%) |  |
|  |  |  |  |  |
| AFP | <200pg/ml | 115 (70.99%) | 76 (73.79%) | 0.621 |
|  | >200pg/ml | 47 (29.01%) | 27 (26.21%) |  |
|  |  |  |  |  |
| Histologic grade | 1+2 | 131 (64.22%) | 91 (62.33%) | 0.718 |
|  | 3+4 | 73 (35.78%) | 55 (37.67%) |  |
|  |  |  |  |  |
| Vascular invasion | No | 129 (70.11%) | 67 (57.76%) | 0.033* |
|  | Micro | 50 (27.17%) | 40 (34.48%) |  |
|  | Macro | 5 (2.72%) | 9 (7.76%) |  |
|  |  |  |  |  |
| Pathologic stage | 1+2 | 109 (57.67%) | 55 (41.67%) | 0.005** |
|  | 3+4 | 80 (42.33%) | 77 (58.33%) |  |

*, The Chi-square statistic is significant at the 0.05 level; **, The Chi-square statistic is significant at the 0.01 level.

**Supplementary Table S3.** Correlation between CD133 and CXCL3 mRNA Expression in TCGA Database

|  | CXCL3 | | | *p* Value |
| --- | --- | --- | --- | --- |
|  |  | Low | High |  |
|  |  | Count | Count |  |
| CD133 | Low | 143(69.27%) | 50(65.33%) | 0.000** |
|  | High | 69(30.73%) | 109(34.67%) |  |

**, the Chi-square statistic is significant at the 0.01 level.

**Supplementary Methods**

**Supplementary Table S4.** Primer for Chip-PCR

| Name | Primer Sequence |
| --- | --- |
| CXCL3-F  CXCL3-R | 5`-CAGCTCTTTCCTCCAACCCT-3`  5`-GAAATTCCCGGAGCTCCAGA-3` |

F, forward primer; R, reverse primer; RT, reverse-transcription primer

**Supplementary Table S5.** Primers for qRT-PCR

| Name | Primer sequence |
| --- | --- |
| CD133-F  CD133-R  CXCL3-F  CXCL3-R | 5`-TGGCAACAGCGATCAAGGAGAC-3`  5`-TCGGGGTGGCATGCCTGTCATA-3`  5`-TCCGTGGTCACTGAACTGCG-3`  5`-AGTTGGTGCTCCCCTTGTTCA-3` |

F, forward primer; R, reverse primer; RT, reverse-transcription primer

**Supplementary Table S6.** Antibodies used in this study

| Antibody | Clone, host | Dilution | Company |
| --- | --- | --- | --- |
| **For Western blotting** | | | |
| Ikaros | H-100, rabbit polyclonal | 1:200 | Santa Cruz |
| CD133 | W6B3C1, mouse IgG1 | 1:100 | MACS |
| p-ETS1 | pT38, rabbit polyclonal | 1:300 | Invitrogen |
| ETS1 | H-150, rabbit polyclonal | 1:500 | CST |
| CXCL3 | rabbit polyclonal | 1:300 | Abcam |
| β-actin | AC-15, mouse mAb | 1:20,000 | Sigma |
| Secondary antibody | HRP conjugated goat anti-rabbit IgG | 1:3,000 | Sigma |
| Secondary antibody | HRP conjugated goat anti-mouse IgG1 | 1:3,000 | Santa Cruz |

**Supplementary Table S7.** Cloned primer sequences

| Name | Primer sequence |
| --- | --- |
| CXCL3-promoter-F  CXCL3-promoter-R | 5`-GGATGGTACCAACATTTCAGAGGGAGGAAGG-3`  5`-AAGAAAGCTTCCACCAGGAGCAGGAGCA-3` |

F, forward primer; R, reverse primer; RT, reverse-transcription primer

**Supplementary Table S8.** shRNA sequences

| Name | Sequence |
| --- | --- |
| CXCL3-sh | ATCCAAAGTGTGAATGTAA |

NOTE. The threshold value of the fold change used to screen for decreased mRNA expression was ≥ 1.5.

**Supplementary Table S9. CXCL3 promoter sequence**

| **>CXCL3 promoter**  -717 AACATTTCAGAGGGAGGAAGGACGTTATGCAGGATACAAAAGAAGAGGTCATGTTATACA  -657 GCCCTGGCTTCCACGGACACTAACACTGAATTCAAATTTTGACACTGATAATCTGTTGCC    -597 ACCAAATGGAAAACGTAAACAAGGTATTCTAAGTGTGATTAGAGAATATGCAAAACAAGG  -537 AACAAGTAGAACATTCTTCTCTGGAATCCGAGACGATGGCTGTACTTTCACAGAGAGCAT  -477 GATGTTAGATGTACATGAAATAACGCTAAACCGAAAATGAGAGAGGCAGAGACCGGGAGG  -417 TTAACATAGAGGATAGACTATATAGAGAGAGGATAGCTGAGGGAAAACTCGCCTGTCTCC  -357 GGGTCCCCAGCAAATCTGATAACTAAGGAGACAAAGCTCTCTTCCTCTAAAGAAGTCGTG  -297 CCTTTCCTGTCCTGGTTCTCACGGGTCCCCCCAGCTCTTTCCTCCAACCCTACCCCGTAC  -237 GCGGGGGGTCATCGGGGACCTAAGGTCCCCCCTCACAGGCTGTATCTTCAGCGAGGTGGA  -177 CTCACTGCCTCTCCAGGAATTTGGGGCAGAAAATGAATATCCCAAAGTCCCAGAGTGCAC  -57 GACTCCACCCCGGGGGCGGGGCCGTCGCCTTCCTTCGGGACTCCGGATCGATCTGGAGCT  Transcriptional initiation site  117 CCGGGAATTTCCCTGGCCCGGCCGCTCCGGGCTTTCCAGTCTCAACCATGCATAAAAAGG  177 GTTCGCCGATCTTGGGGAGCCACACAGCCCGGGTCGCAGGCACCTCCCCGCCAGCTCTCC  237 CGCTTCTCGCACAGCTTCCCGACGCGTCTGCTGAGCCCCATGGCCCACGCCACGCTCTCC  295 GCCGCCCCCAGCAATCCCCGGCTCCTGCGGGTGGCGCTGCTGCTCCTGCTCCTGGTGG |
| --- |

**
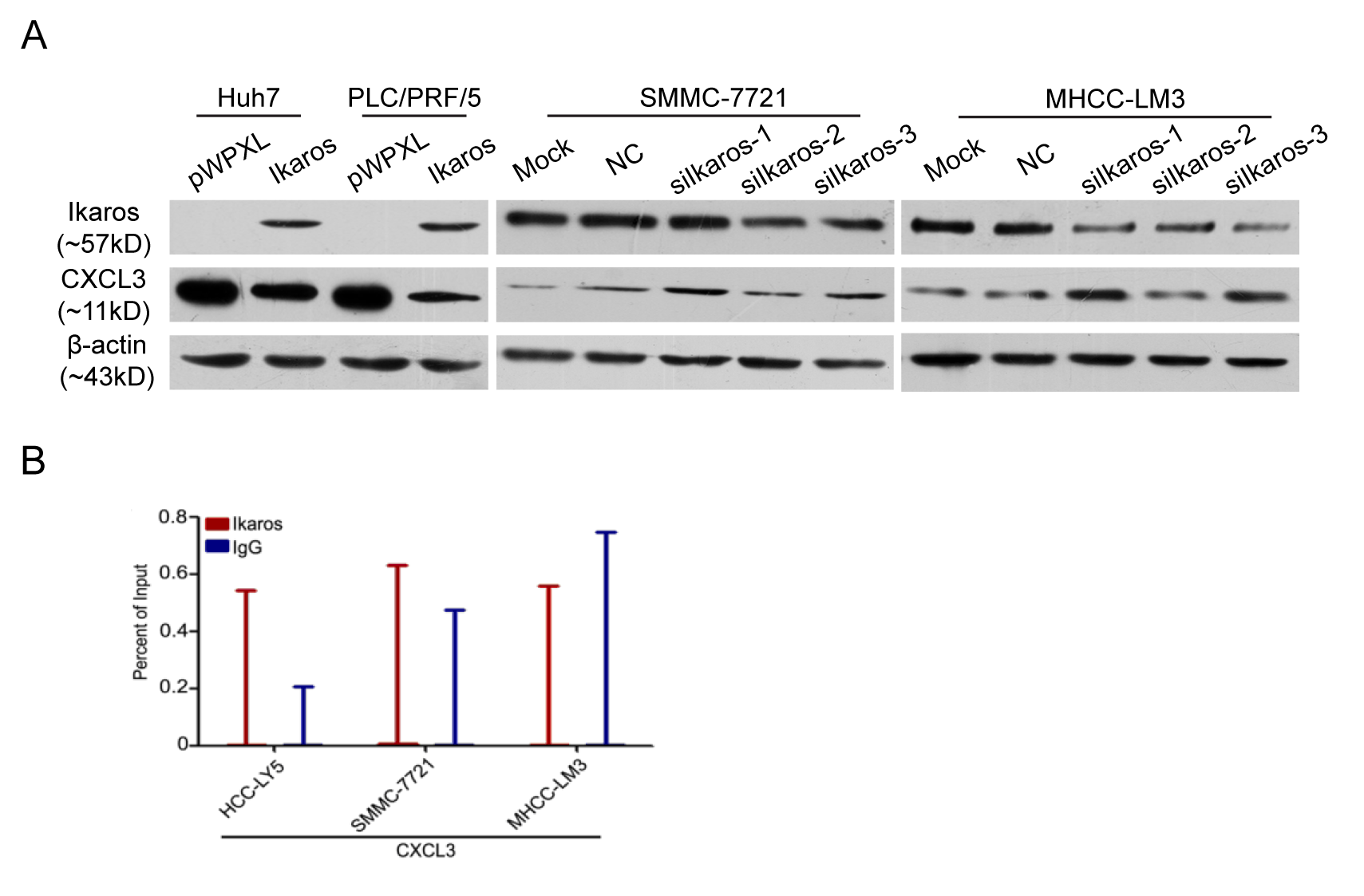
**

**Supplementary Figure S1. CXCL3 is not a target gene of Ikaros protein.** (A) Ikaros protein expression was negatively correlated with CXCL3 expression in HCC cells. (B) ChIP assays and q-PCR were performed to confirm the Ikaros binding with the CXCL3 promoter.

**
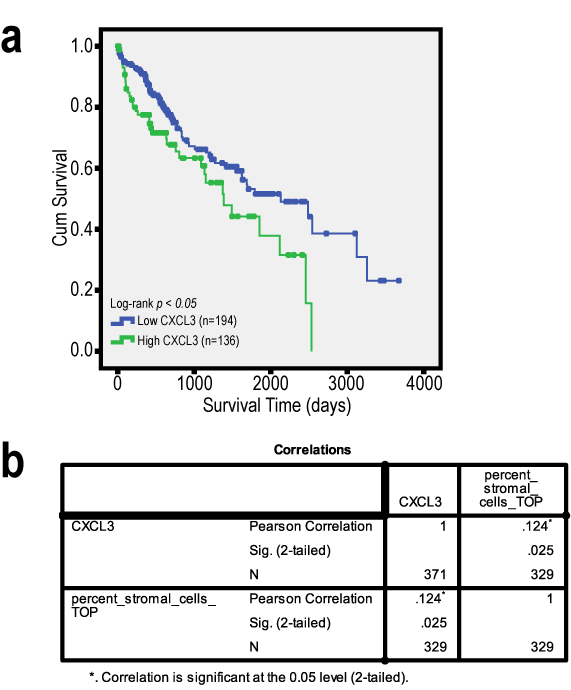
**

**Supplementary Figure S2. CXCL3 mRNA expression positively correlated with poor prognosis of HCC patients in TCGA cohort.** (A) The overall survival analysis of HCC patients with CXCL3 mRNA expression. N=330; Log-rank *p*=0.015. (B) Pearson correlation analysis result showed that CXCL3 expression positively correlated with the percentage of stromal cells at the top of HCC tissue.


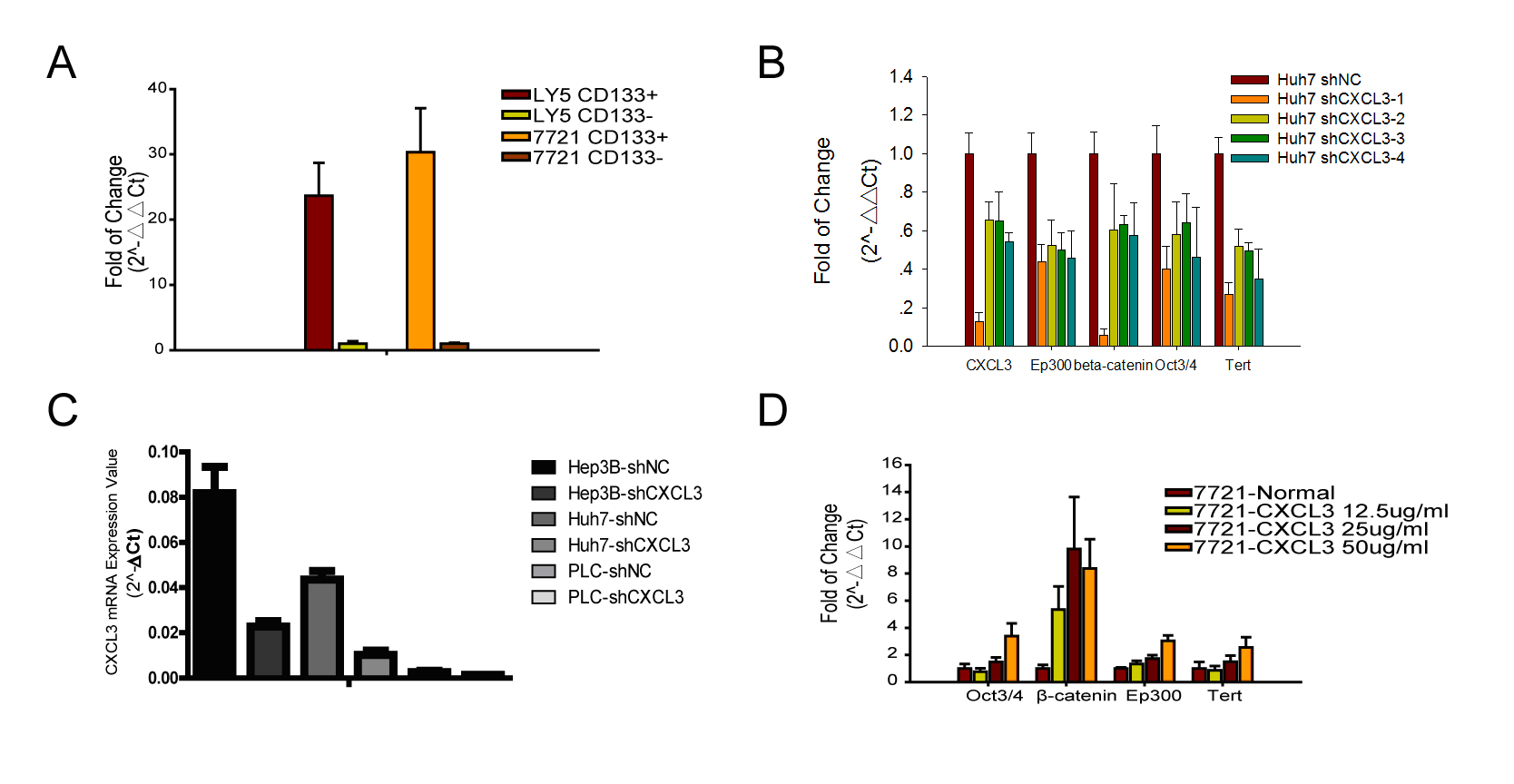


**Supplementary Figure S3. CXCL3 contributed to stemness-related genes expression in HCC cells.** (A) CD133 mRNA expression was overexpressed in the sorted CD133^+^ cell population in HCC-LY5 and SMMC-7721 cell. (B) CXCL3 knockdown downregulated stemness-related genes including Oct3/4, β-catenin, Ep300 and Tert expression in Huh7 cells. (C) CXCL3 mRNA expression was successfully knockdown in HCC cells. (D) CXCL3 treatment upregulated stemness-related genes including Oct3/4, β-catenin, Ep300 and Tert expression in SMMC-7721 cells.


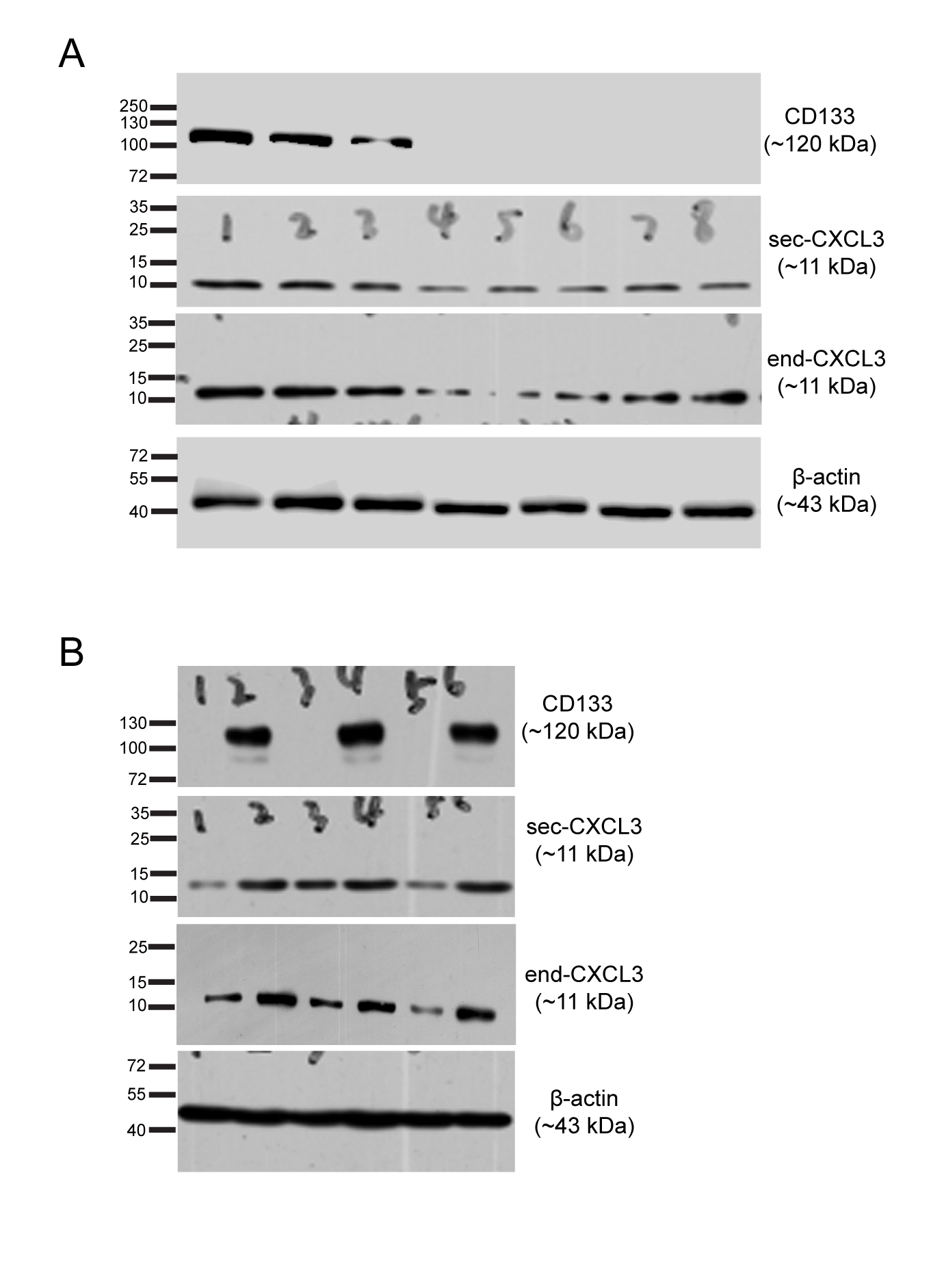


**Supplementary Figure S4.** Full-length images of Western blots results in Figure 1.


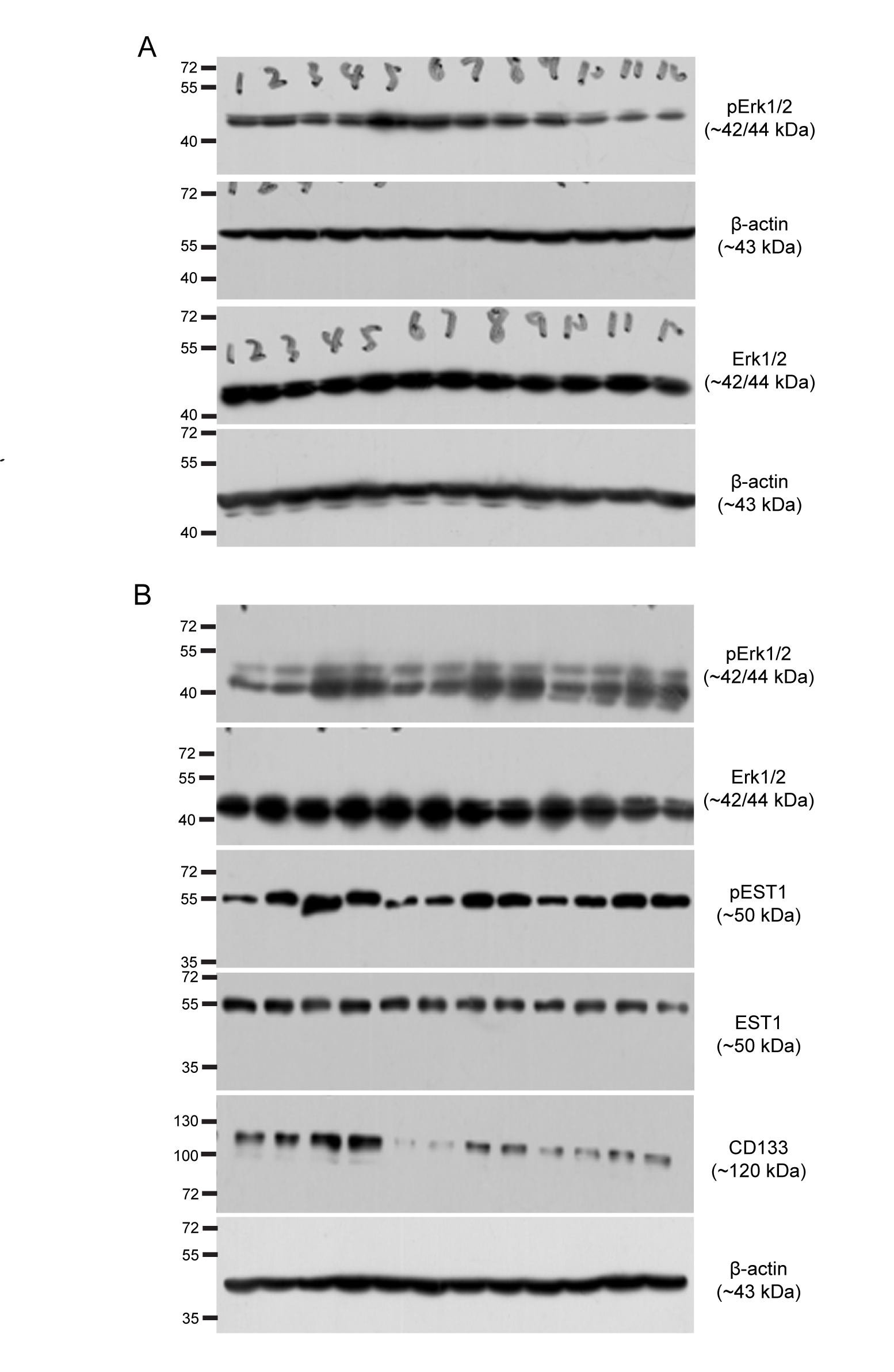


**Supplementary Figure S5.** Full-length images of Western blots results in Figure 3.


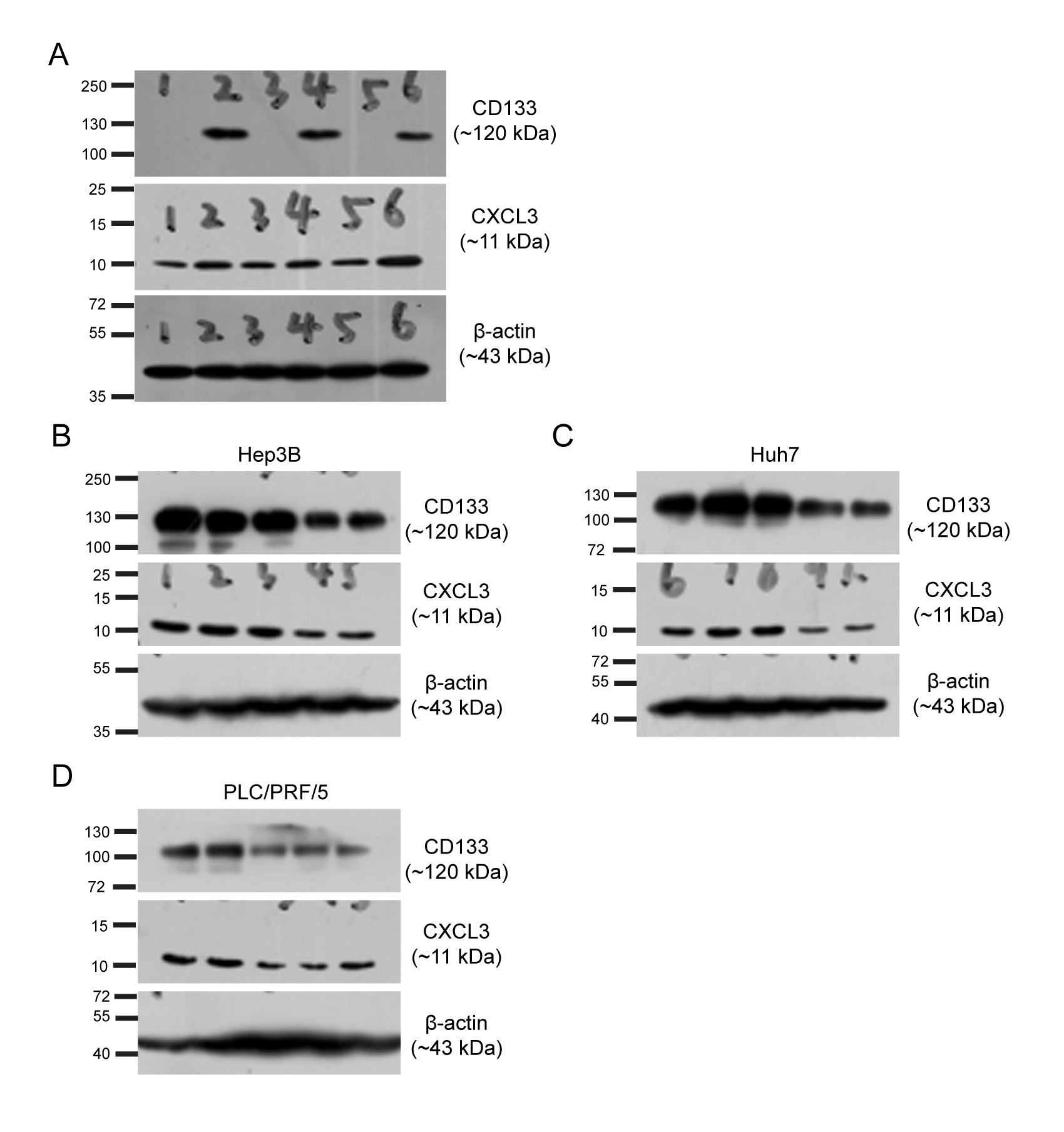


**Supplementary Figure S6.** Full-length images of Western blots results in Figure 4.


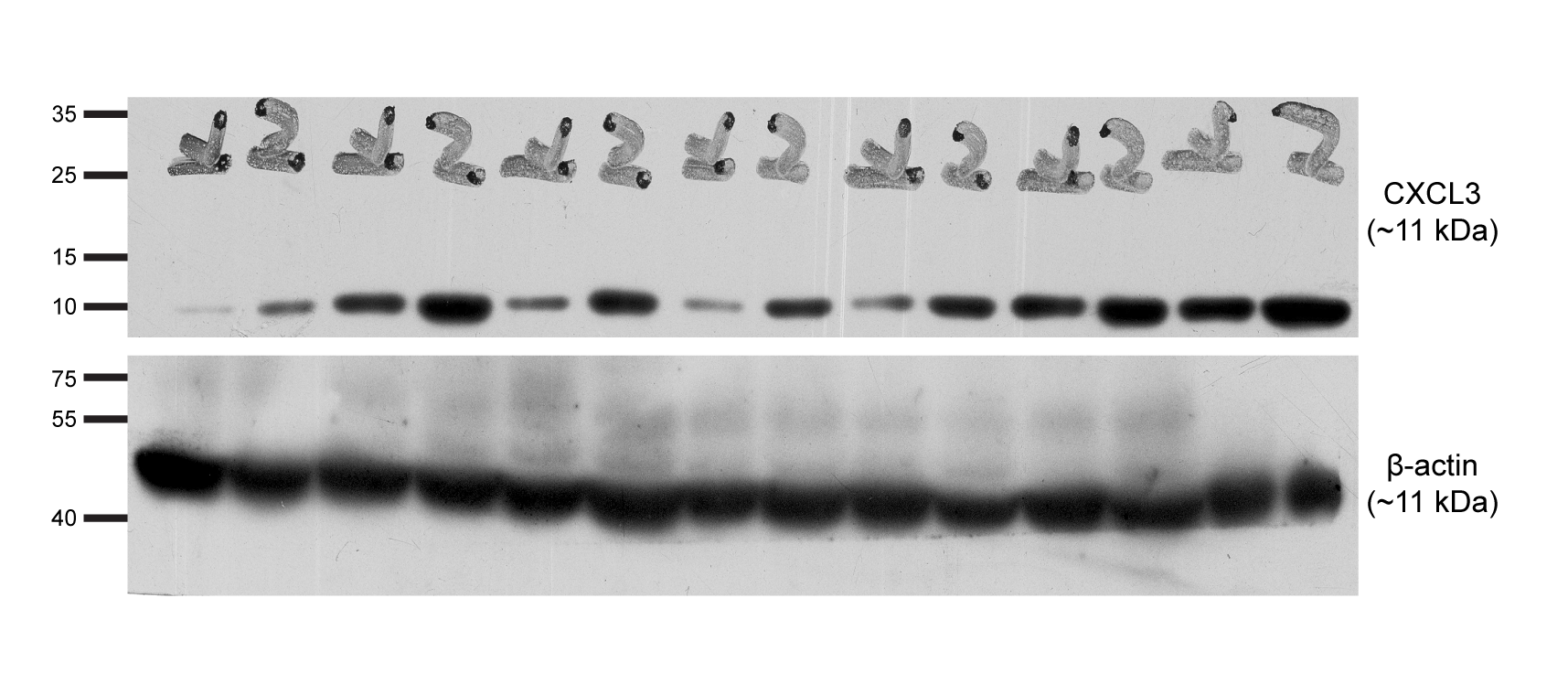


**Supplementary Figure S7.** Full-length images of Western blots results in Figure 5.
